# Supplementary material for: Tissue-Specific Requirement for the GINS Complex During Zebrafish Development
Source: Front Cell Dev Biol. 2020 May 28;8:373. doi: 10.3389/fcell.2020.00373 (PMC7270345; doi:10.3389/fcell.2020.00373)
Supplement: Supplementary file 1 [file Image_1.pdf]

Supplementary Material

1 Supplementary Materials and Methods

1.1 Neutral Red staining

Embedding and sectioning of 5 dpf larvae was performed as described in the general Materials and Methods section. Sections were stained in freshly prepared 0.001% Neutral Red (N4638, Sigma-Aldrich) solution for 5 min, washed in dH_2O, dried and covered.

1.2 Alcian Blue staining

Larvae were fixed in 4% PFA, incubated in 50% ethanol for one day and transferred to 100% ethanol. After 24 hours, the staining solution was added (0.02% Alcian Blue (A5268, Sigma-Aldrich), 29.8% acetic acid, 70% ethanol)

1.3 RT-PCR analysis

Embryos at 2 dpf stage from a *u773* in-cross were selected based on their phenotype. Total RNA was isolated from 10-10 *u773* embryos and siblings, using the TRIzol™ method, according to the manufacturer's protocol (15596018, Invitrogen). cDNA synthesis was performed using the SuperScript™ III First-Strand Synthesis System (18080051, Invitrogen). Primers used to amplify *gins2* were described in the 4.2 and 4.3 sections of the main Methods section. Primers used to amplify *ef1a* and *actb* were as follows: ef1a-F – 5'-ACCGGCCATCTGATCTACAA-3', ef1a-R – 5'-CAATGGTGATACCACGCTCA-3', actb-F – 5'-CGAGCAGGAGATGGGAACC-3', actb-R – 5'-CAACGGAAACGCTCATTGC-3'.

2 Supplementary Figures

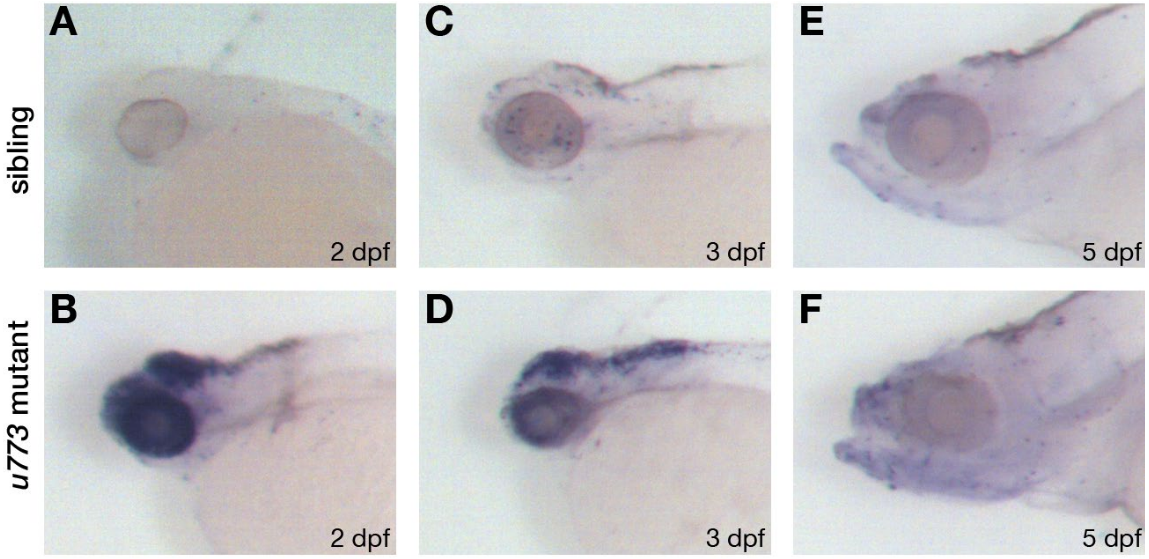

Supplementary Figure 1. Transient apoptosis can be observed in *u773* mutant embryos**.**

A-F) Lateral views of TUNEL-stained wildtype (A,C,E) and *u773* mutant (B,D,F) embryos at stages indicated.

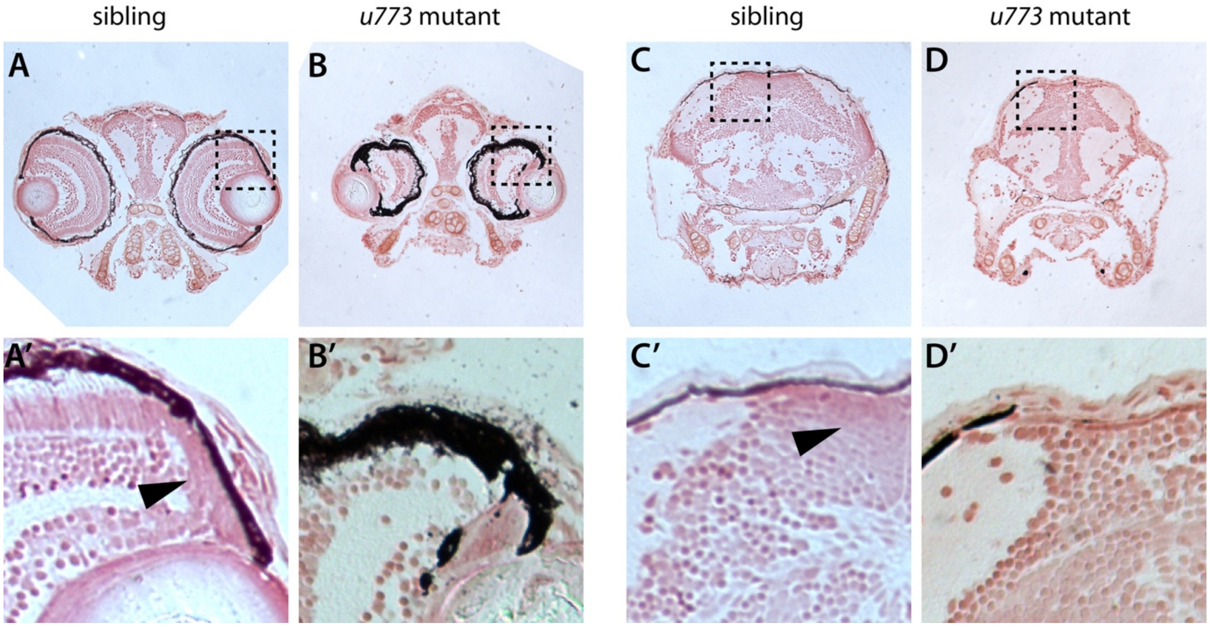

Supplementary Figure 2. Histological analysis of 5 dpf *u773* mutant larvae.

A-B') Transverse sections of wildtype and mutant zebrafish larvae through the eyes. A' and B' show magnified views of the boxes in A and B. Note that the progenitor zone in the eye, denoted by the arrowhead in the wildtype larvae (A') is missing in the *u773* mutants (B').

C-D') Transverse sections of wildtype and mutant zebrafish larvae through the OT areas. C' and D' show magnified views of the boxes in C and D. Note that the progenitor zone in the dorsomedial region of the tectum, denoted by the arrowhead in the wildtype larvae (C') is missing in the *u773* mutants (D').

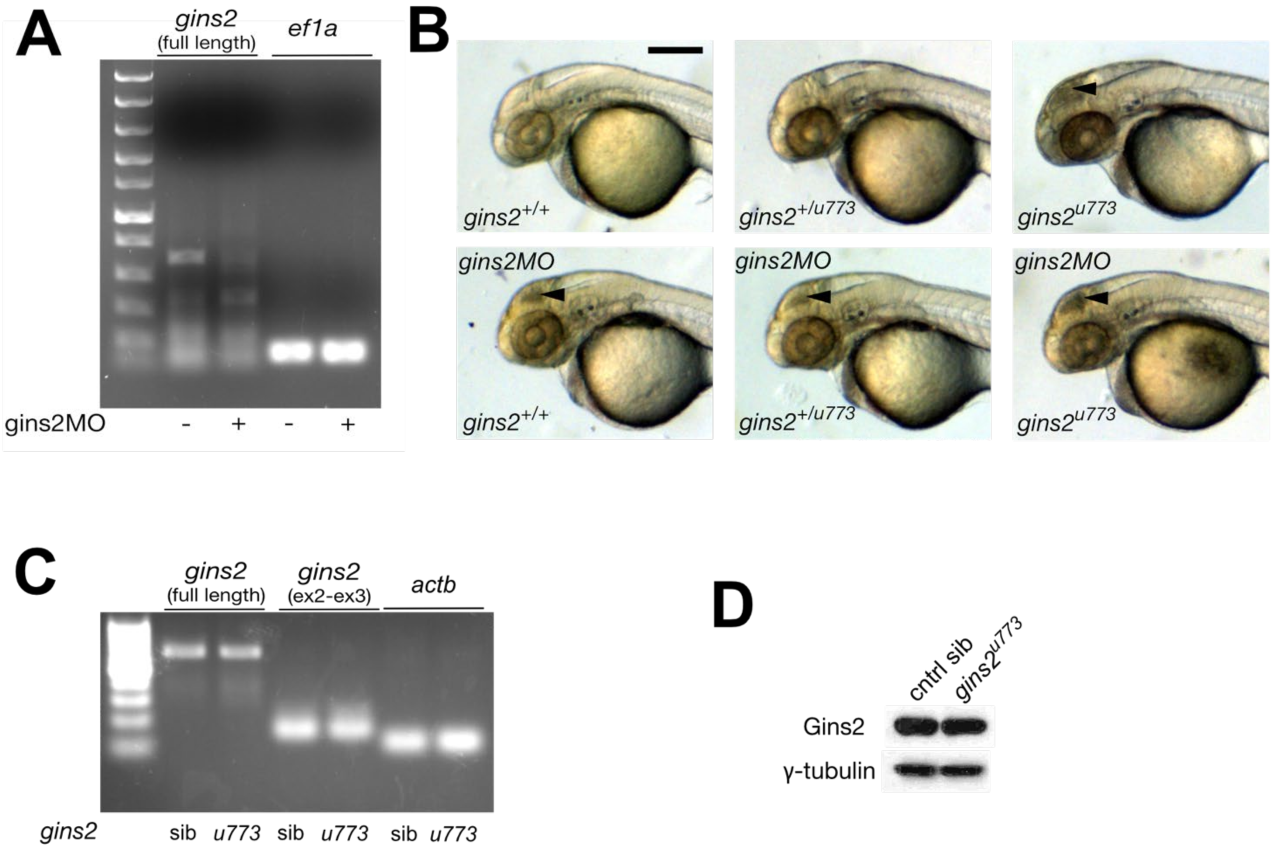

Supplementary Figure 3. *gins2*MO phenocopies the missense *gins2*^{u773} mutation

A) RT-PCR on 2 dpf *gins2*MO injected samples suggests that mis-spliced *gins2* mRNA undergoes non-sense mediated decay (NMD).

B) Phenotype of *gins2*^{u773} mutant embryos and their siblings injected with 1 mM *gins2*MO. Arrowheads point to apoptotic cells in the OT. (Scale bar: 250 μm.)

C) RT-PCR of the full length *gins2* and an exon2-exon3 fragment of *gins2* from 2 dpf *gins2*^{u773} mutant and siblings; *actb* is used as a control.

D) Western blot analysis of protein lysates from 2 dpf control and *gins2*^{u773} embryos probed with Gins2 and γ-tubulin antibodies.

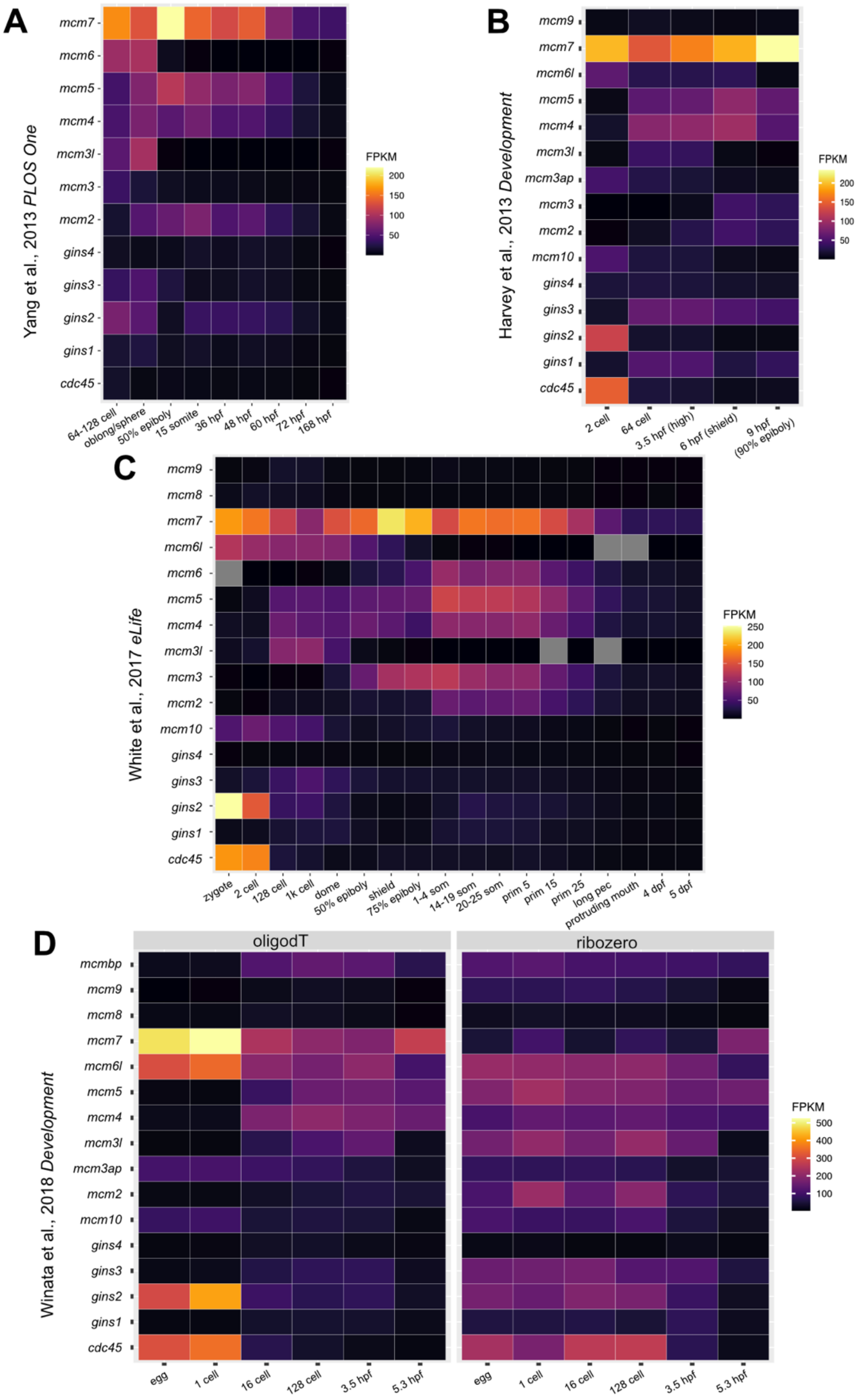

Supplementary Figure 4. The expression of CMG genes during early zebrafish development.

A-D) Fragments per kilobase of exon model per million reads mapped (FPKM) value of different genes at given stages as shown by the datasets in the respective papers (Harvey et al., 2013; White et al., 2017; Winata et al., 2018; Yang et al., 2013).

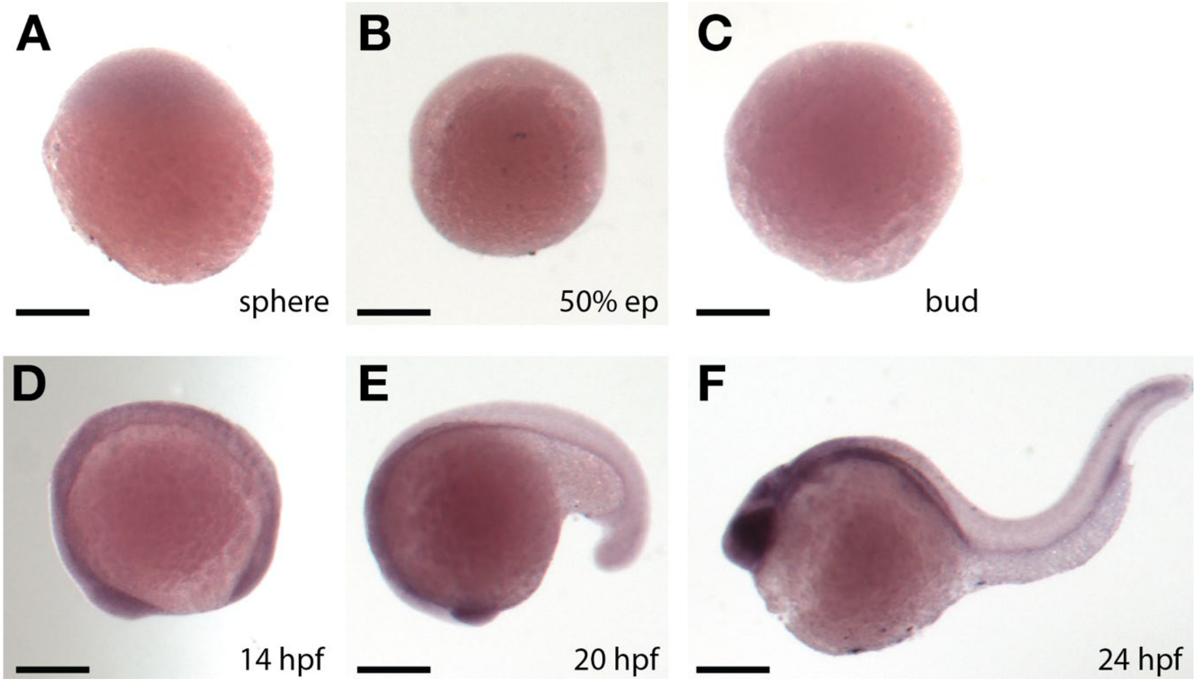

Supplementary Figure 5. The expression of *gins1* during zebrafish development.

A-F) Lateral views of the wildtype embryos assessed for *gins1* expression with whole mount *in situ* hybridization at the indicated stages. Scale bars: 150 μm.

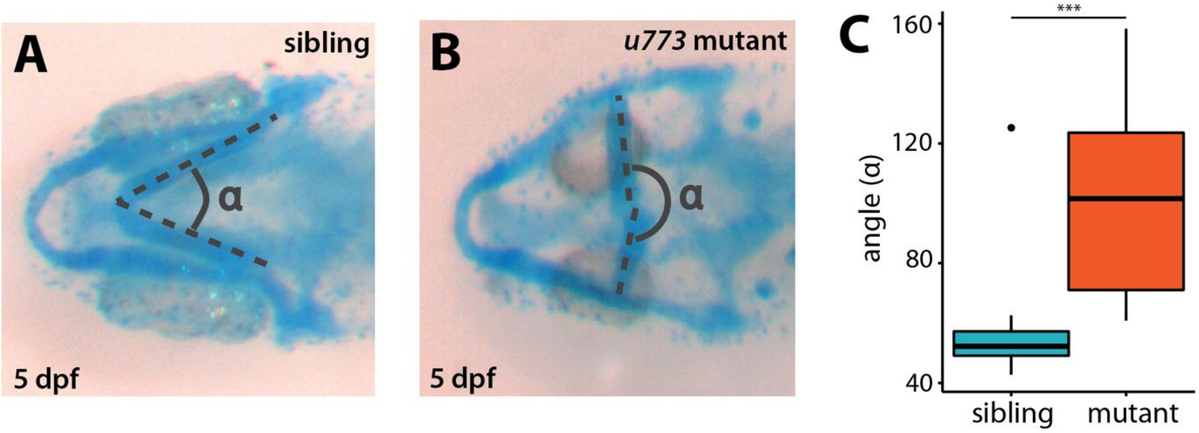

Supplementary Figure 6. Jaw cartilage development is severely impaired in *u773* mutants.

A,B) Alcian Blue staining of the jaw cartilage in 5 dpf wildtype (A) and *u773* mutant (B) larvae.

C) The angle of ceratohyals of 5 dpf mutant larvae stained is significantly different compared to wildtype (n=19) ($p<0.001$).

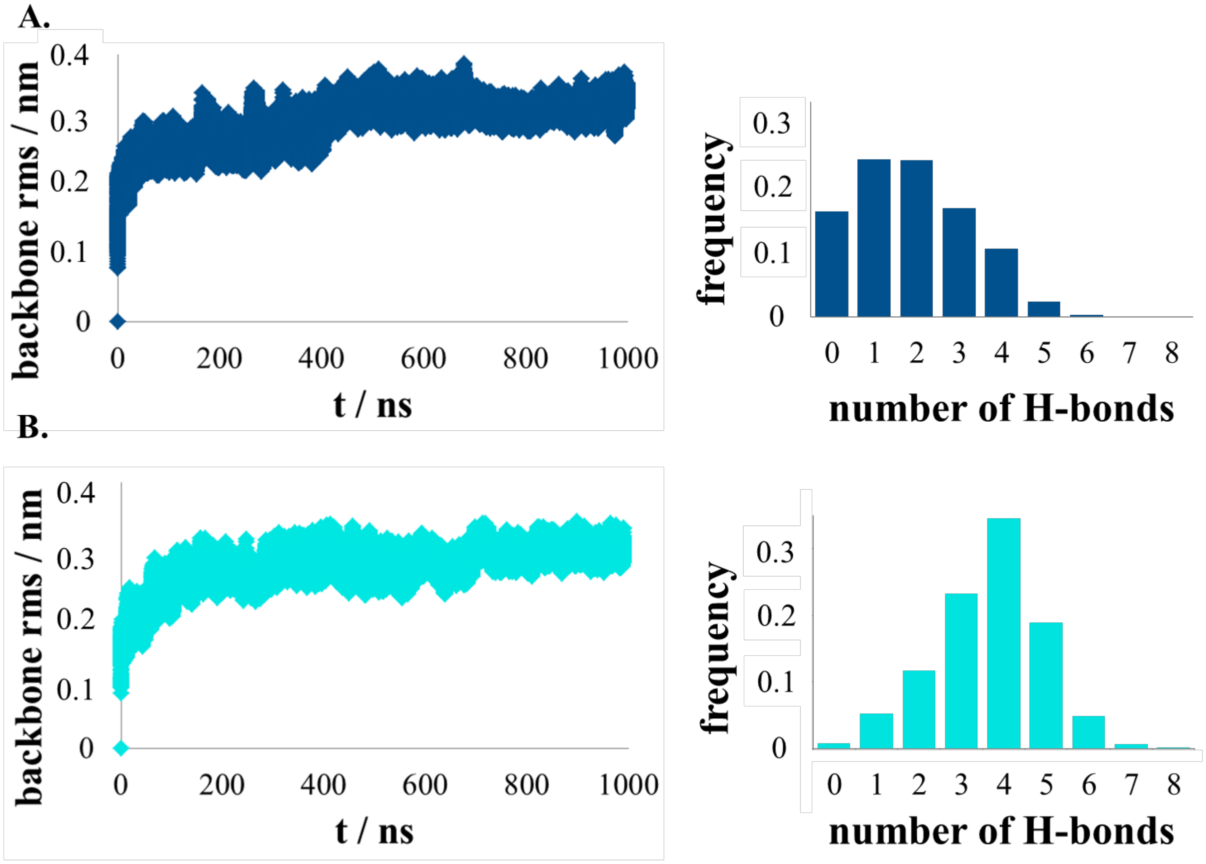

Supplementary Figure 7. The root-mean-square deviation of the protein backbone and the number of H-bonds formed between the neighboring regions of Gins2 (residues 48-58) and Gins4 (64-74) in case of the wildtype (A) and the Gins2^{L52P} (B) tetramer.

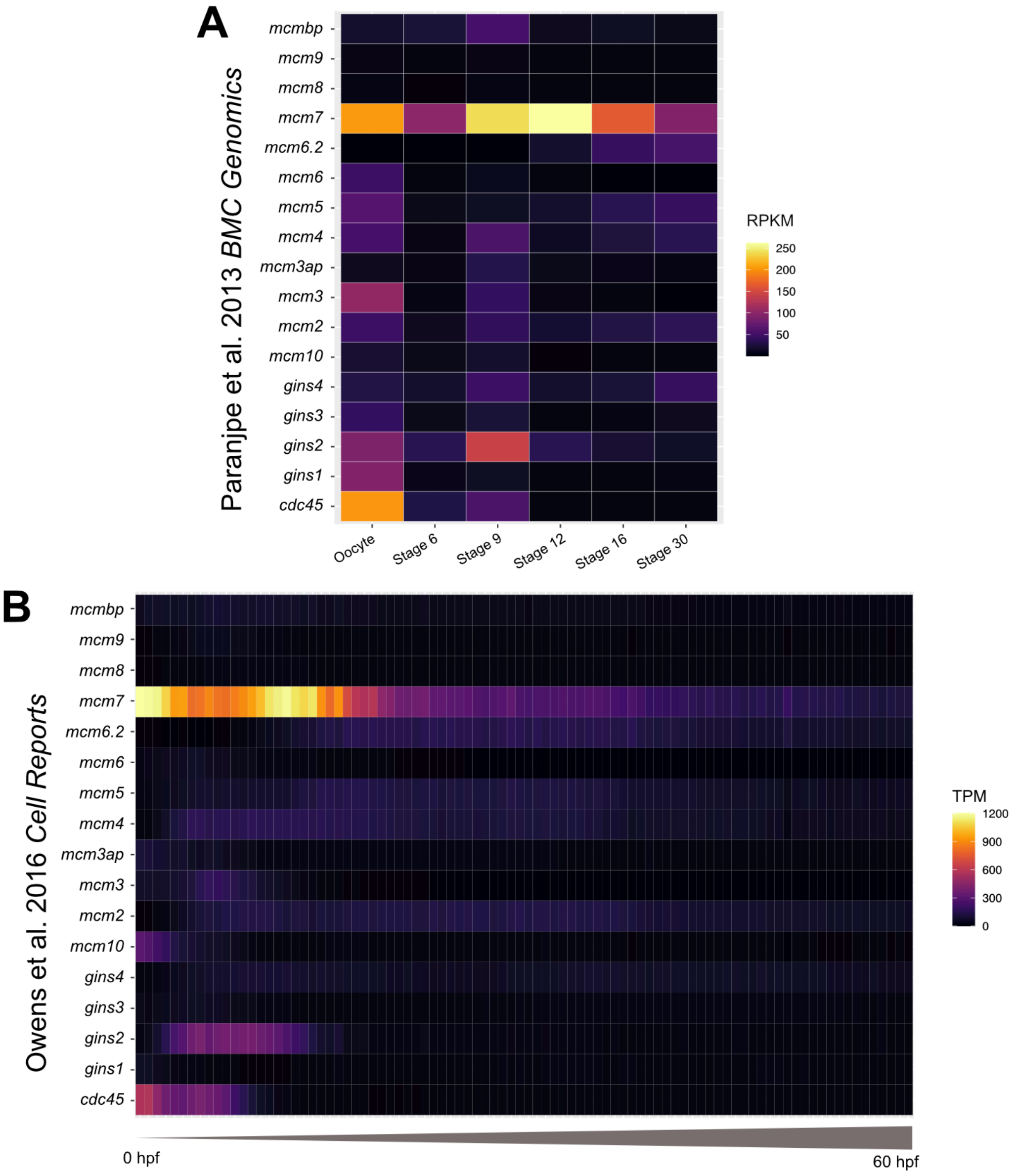

Supplementary Figure 8. The expression of CMG genes during early *Xenopus tropicalis* development.

A) Reads per kilobase of exon model per million reads mapped (RPKM) values of different genes at given stages as shown by the dataset in (Paranjpe et al., 2013).

B) Transcripts per million (TPM) values of different genes at given stages as shown by the dataset in (Owens et al., 2016).

3 Supplementary references

Harvey, S. A., Sealy, I., Kettleborough, R., Fényes, F., White, R., Stemple, D., et al. (2013). Identification of the zebrafish maternal and paternal transcriptomes. *Development* 140, 2703–2710. doi:10.1242/dev.095091.

Owens, N. D. L., Blitz, I. L., Lane, M. A., Patrushev, I., Overton, J. D., Gilchrist, M. J., et al. (2016). Measuring Absolute RNA Copy Numbers at High Temporal Resolution Reveals Transcriptome Kinetics in Development. *Cell Rep* 14, 632–647. doi:10.1016/j.celrep.2015.12.050.

Paranjpe, S. S., Jacobi, U. G., van Heeringen, S. J., and Veenstra, G. J. C. (2013). A genome-wide survey of maternal and embryonic transcripts during Xenopus tropicalis development. *BMC Genomics* 14, 762–17. doi:10.1186/1471-2164-14-762.

White, R. J., Collins, J. E., Sealy, I. M., Wali, N., Dooley, C. M., Digby, Z., et al. (2017). A high-resolution mRNA expression time course of embryonic development in zebrafish. *Elife* 6, 1328. doi:10.7554/eLife.30860.

Winata, C. L., Łapiński, M., Pryszcz, L., Vaz, C., Bin Ismail, M. H., Nama, S., et al. (2018). Cytoplasmic polyadenylation-mediated translational control of maternal mRNAs directs maternal-to-zygotic transition. *Development* 145, dev159566. doi:10.1242/dev.159566.

Yang, H., Zhou, Y., Gu, J., Xie, S., Xu, Y., Zhu, G., et al. (2013). Deep mRNA sequencing analysis to capture the transcriptome landscape of zebrafish embryos and larvae. *PLoS ONE* 8, e64058. doi:10.1371/journal.pone.0064058.
